# Supplementary material for: Omicron infection following vaccination enhances a broad spectrum of immune responses dependent on infection history
Source: Nat Commun. 2023 Aug 21;14:5065. doi: 10.1038/s41467-023-40592-4 (PMC10442364; doi:10.1038/s41467-023-40592-4)
Supplement: Supplementary file 1 — Supplementary Information [file 41467_2023_40592_MOESM1_ESM.pdf]

SUPPLEMENTARY INFORMATION

Table S1. Antibodies used for multiparameter flow cytometry (Newcastle)

Reagent (clone)	Source	Identifier	Titration	Reference Control	Events acquired for reference control
CD62L-BB515 (SK11)	Becton Dickinson	565037	1/100	Cells	50,000
CCR7-PerCP (G043H7)	BioLegend	353242	1/20	Cells	70,000
CD57-PerCP_ Cy5.5 (HNK4)	BioLegend	359622	1/100	Cells	60,000
CD38-PerCP_ eFluor_710 (HB7)	ThermoFisher	46-0388-42	1/50	Cells	50,000
CD25-PE_Cy7 (M-A251)	Becton Dickinson	557741	1/50	Cells	50,000
CD45RA-Alexa Fluor 647 (HI100)	BioLegend	304154	1/50	Cells	50,000
CCR5-Alexa Fluor 700 (HEK_1_85a)	BioLegend	313713	1/100	Cells	100,000
CD69-APC_Cy7 (FN50)	BioLegend	310914	1/100	Cells	50,000
CD3-APC Fire 810 (SK7)	BioLegend	344858	1/200	Cells	30,000
PD-1-BV421 (EH12.1)	Becton Dickinson	562516	1/50	Cells	50,000
CD27-Super Bright 436 (0323)	ThermoFisher	62-0279-42	1/100	Cells	50,000
CD45RO-Pacific Blue (UCHL1)	BioLegend	304216	1/100	Cells	50,000
CCR2-BV480 (LS132.1D9)	Becton Dickinson	747852	1/100	Cells	100,000
CD4-eFluor 506 (RPA-T4)	ThermoFisher	69-0049-42	1/200	Cells	30,000

HLA-DR-BV570 (L243)	BioLegend	307638	1/50	Cells	50,000
CD28-BV605 (L293)	Becton Dickinson	742527	1/50	Cells	50,000
CD95-BV650 (DX2)	Becton Dickinson	740589	1/100	Cells	50,000
KLRG1-BV711 (2F1)	BioLegend	138427	1/50	Cells	80,000
CXCR6-BV786 (13B 1E5)	Becton Dickinson	743602	1/20	Cells	150,000
CD19-BUV395 (SJ25C1)	Becton Dickinson	563549	1/100	Cells	50,000
CD127-BUV737 (HIL-7R-M21)	Becton Dickinson	612794	1/50	Cells	80,000
CD8-BUV805 (SK1)	Becton Dickinson	612889	1/200	Cells	30,000
**Surrogates**					
CD3-APC (UCHT1)	BioLegend	300412	1/200	Cells	30,000
CD3-PE (UCHT1)	BioLegend	300408	1/200	Cells	30,000

Table S2. Antibodies used for multiparameter flow cytometry (Sheffield)

Reagent (clone)	Source	Identifier	Titration	Reference Control	Events acquired for reference control
CD56-Biotin (HCD56)	Biolegend	318320	1/100	Cells	20,000
CD19-Biotin (HIB19)	Biolegend	302204	1/200	Cells	20,000
Streptavidin AF532	ThermoFisher	S11224	1/200	Cells	20,000
Zombie NIR	Biolegend	423106	1/2000	Cells	20,000
CD3-APC/Fire 810 (SK7)	Biolegend	344858	1/200	Cells	20,000
CD4-Spark NIR 685 (SK3)	Biolegend	344657	1/400	Cells	20,000
CD8-BV750 (SK1)	Biolegend	344756	1/100	Cells	20,000
CCR7-PE-Cy5 (G043H7)	Biolegend	353272	1/20	Cells	20,000
CD45R0-BV711 (UCHL1)	BD Biosciences	563722	1/20	Cells	20,000
CD28-BV605 (L293)	BD Biosciences	742527	1/50	Cells	20,000
CD27-BV421 (M-T271)	BD Biosciences	562513	1/50	Cells	20,000
CD95-BV650 (DX2)	BD Biosciences	740589	1/200	Cells	20,000
Granzyme B-Pacific Blue (GB11)	Biolegend	515408	1/200	Cells	20,000
CD62L-BB700 (SK11)	BD Biosciences	745995	1/50	Cells	20,000

CD137-PE/ Dazzle 594 (4B4-1)	Biolegend	309826	1/100	Cells	20,000
CXCR6-BV786 (13B 1E5)	BD Biosciences	743602	1/50	Cells	20,000
HLA-DR-BV570 (L243)	Biolegend	307638	1/50	Cells	20,000
CD69-APC Cy7 (FN50)	Biolegend	310914	1/50	Cells	20,000
CD25-PE-Cy7 (M-A251)	BD Biosciences	557741	1/20	Cells	20,000
CCR5-AF700 (HEK/1/85a)	Biolegend	313713	1/100	Cells	20,000
PD-1-VioBright FITC (PD 1.3.1.3.)	Miltenyi Biotec	130-117-681	1/50	Cells	20,000

Table S3. Pentamer/dextramer constructs used for staining the antigen-specific CD3+CD8+ T cells

HLA:peptide construct	Sequence	Epitope origin	Company	Peptide code
PE-HLA-A*02:01	YLQPRTFLL	Spike 269aa-277aa	ProImmune	4339
PE-HLA-A*03:01	KCYGVSPTK	Spike 378aa-386aa	ProImmune	4443
PE-HLA-A*01:01	LTDEMIAQY	Spike 865aa-873aa	ProImmune	custom
PE-HLA-B*57:01	GTITSGWTF	Spike 879aa-888aa	Immudex	WQ06456
PE-HLA-A*01:01	TTDPSSFLGRY	Replicase protein 1637aa-1646aa	ProImmune	4381
PE-HLA-B*07:02	SPRWYFYYL	Nucleocapsid protein 105aa-113aa	ProImmune	4351
APC-HLA-A*02:01	GLCTLVAML	EBV BMLF-1 259aa-1267aa	ProImmune	1
APC-HLA-B*07:02	RPPIFIRRL	EBV EBNA-3A 247aa-255aa	ProImmune	44
APC-HLA-A*03:01	RLRAEQVK	EBV EBNA-3A 603aa-611aa	ProImmune	727
APC-HLA-A*02:01	NLVPMVATV	CMV pp65 495aa-504aa	ProImmune	8

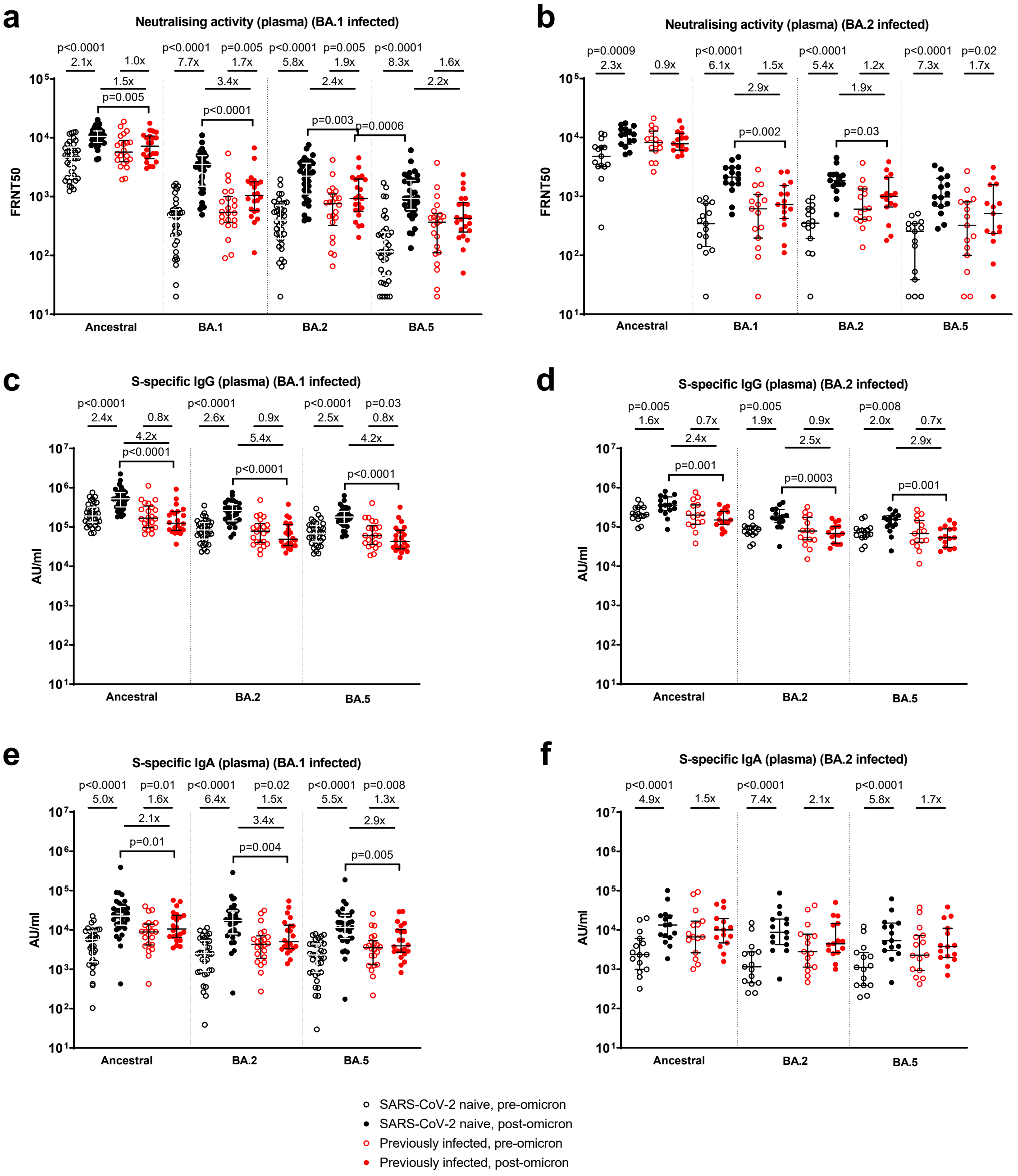

Figure S1. Impact of omicron infection on plasma neutralizing and binding antibodies in SARS-CoV-2-naive and previously-infected individuals, stratified by likely BA.1 or BA.2 infected individuals. Live-virus neutralizing activity of plasma against ancestral, BA.1, BA.2 and BA.5 viruses, expressed as the reciprocal of the dilution showing 50% reduction in focus forming units (FRNT50) in BA.1- (**a**) and BA.2- (**b**) infected individuals; SARS-CoV-2 spike-specific binding IgG in plasma against ancestral, BA.2 and BA.5 spike proteins (AU/mL = arbitrary antibody units/mL in MSD assay) in BA.1- (**c**) and BA.2- (**d**) infected individuals; SARS-CoV-2 spike-specific binding IgA in plasma against ancestral, BA.2 and BA.5 spike proteins (AU/mL = arbitrary antibody units/mL in MSD assay) in BA.1-

(**e**) and BA.2- (**f**) infected individuals; Data shown with median and interquartile range. Median fold-change from pre- to post-infection samples is displayed. Statistical comparisons of pre- and post-infection samples made with two-sided Wilcoxon signed-rank test, and between post-infection levels in previously-infected and SARS-CoV-2 naive individuals using the Mann-Whitney U test. P values are displayed where <0.05. Responses were evaluated in 53 SARS-CoV-2-naive and 37 previously-infected individuals for whom samples were available. Source data are provided as a Source Data file.

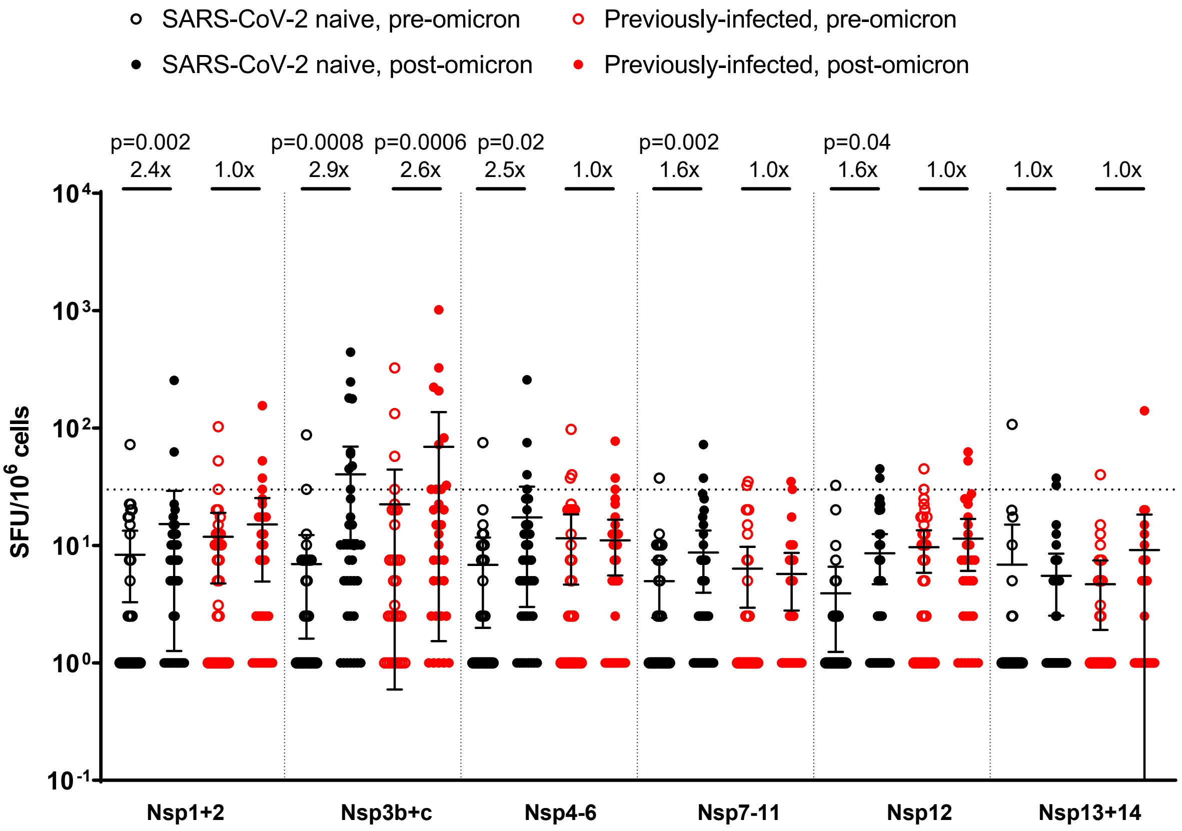

Figure S2. Impact of omicron infection on peptides representing non-structural proteins (NSP). IFN-γ ELISpot responses to overlapping peptide pools representing NSP 1-2, NSP3b+c, NSP4-6, NSP7-11, NSP12 and NSP13+14. Results expressed as spot-forming units per million cells (SFU/10^6). The dashed line represents a positivity threshold of the mean + 2SD of the background response. Data shown with median and interquartile range. Median fold-change from pre- to post-infection samples is displayed. Statistical comparisons of pre- and post-infection samples made with two-sided Wilcoxon signed-rank test. P values are displayed where <0.05. Responses were evaluated in 37 SARS-CoV-2-naive and 32 previously-infected individuals for whom samples were available.

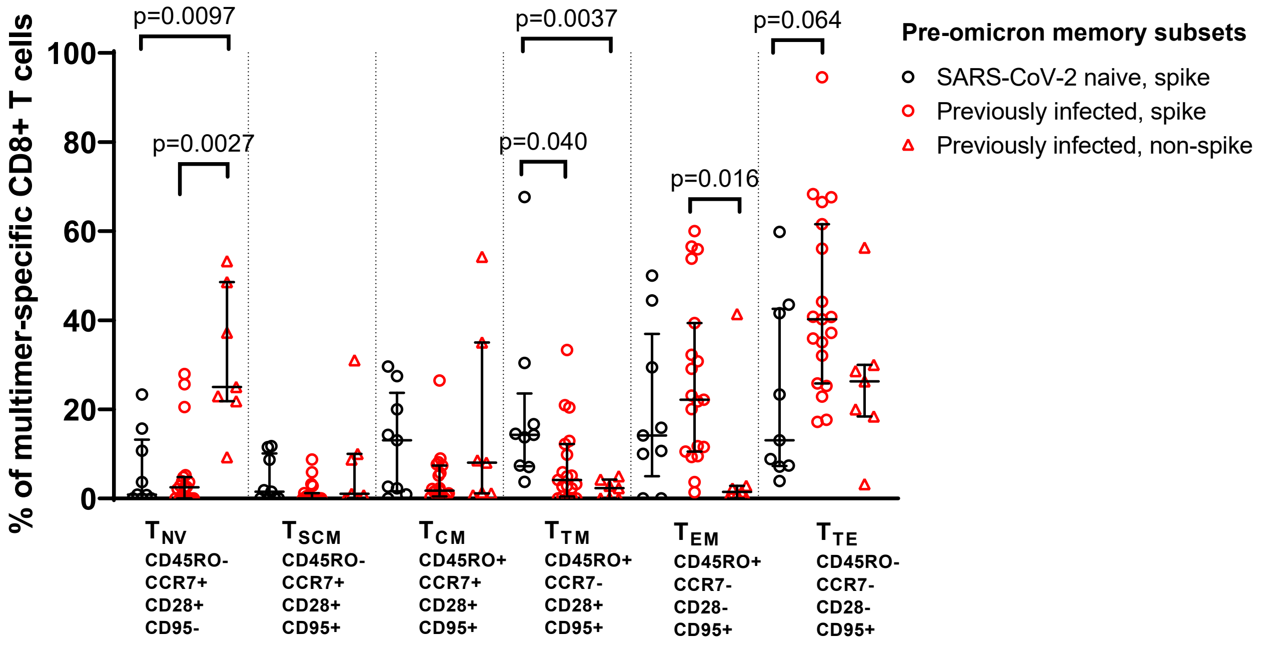

Figure S3. Epitope-specific CD8+ T cells phenotype following 3rd BNT162b2 mRNA vaccine dose but prior to omicron infection. Memory phenotypes of SARS-CoV-2-specific CD8+ T cells prior to omicron infection (pooled data from Sheffield and Newcastle). Shown are 9 spike-specific populations from 8 SARS-CoV-2 naive individuals, 19 spike-specific populations from 14 previously-infected individuals, and 8 non-spike populations from 7 previously-infected individuals. Data shown with median and interquartile range. Statistical comparisons between different multimer-specific populations were performed using Kruskal-Wallis test and Dunn's post-hoc test for multiple pairwise comparisons. P values >0.05 unless displayed. T_{NV} = naive T cells, T_{SCM} = stem cell memory T cells, T_{CM} = central memory T cells, T_{TM} = transitional memory T cells, T_{EM} = effector memory T cells, T_{TE} = terminal effector T cells. Source data are provided as a Source Data file.

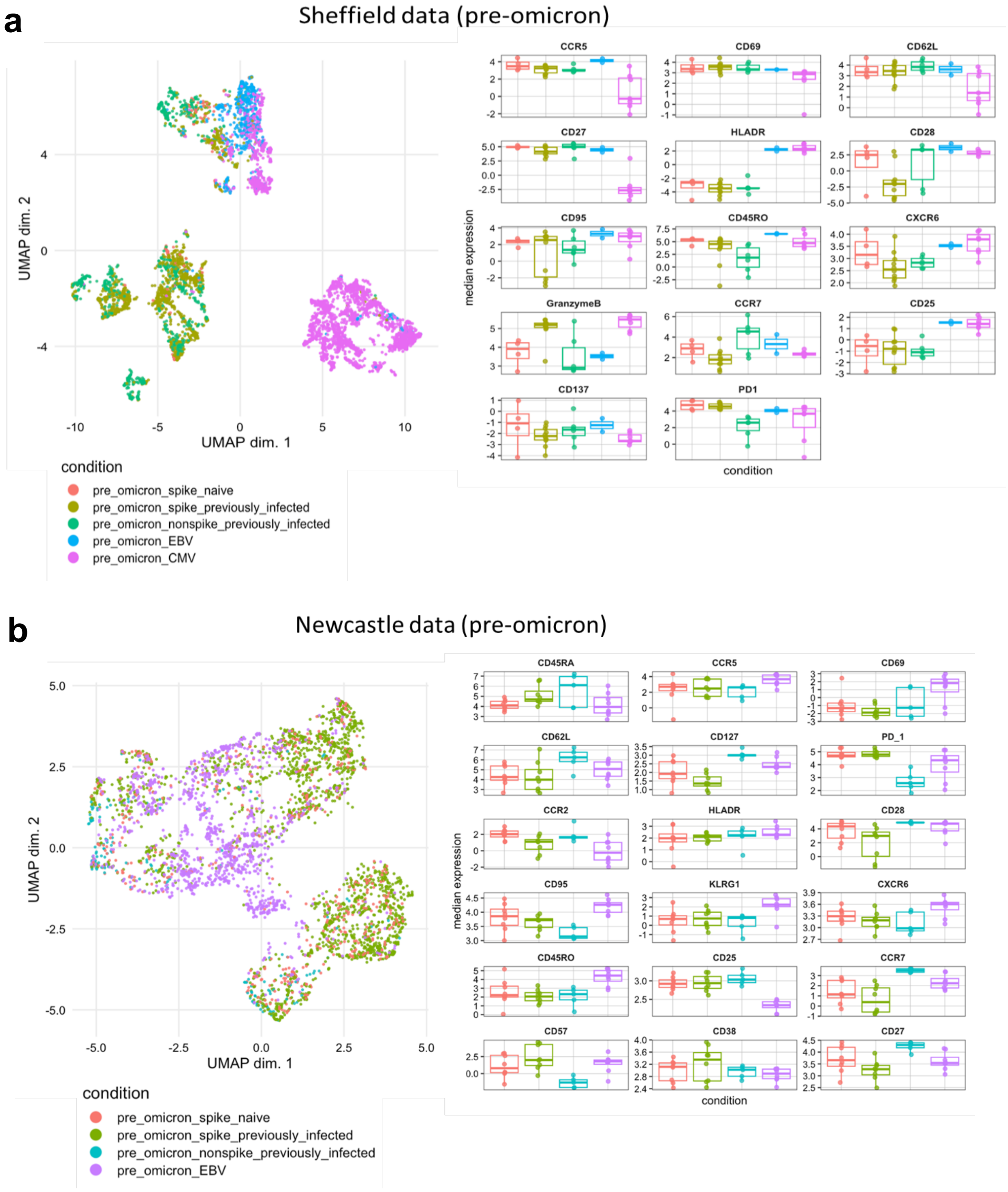

Figure S4. Phenotype of epitope-specific CD8+ T cell populations after 3rd mRNA vaccine dose but prior to omicron infection, characterized using multi-dimensional flow cytometry. Uniform Manifold Approximation and Projection (UMAP) plots and marker expression levels showing multimer-specific cell clusters from SARS-CoV-2 spike-, SARS-CoV-2 non-spike-, EBV- and CMV-specific CD8+ T cells prior to omicron infection, using expression of (**a**) 14 markers (Sheffield panel), shown are data from spike-specific

populations in 4 naïve and 10 previously-infected individuals, non-spike-specific populations in 6 previously-infected individuals, EBV-specific populations in 2 individuals, and CMV-specific populations in 7 individuals; (**b**) 18 markers (Newcastle panel), shown are data from spike-specific populations in 12 naïve and 11 previously-infected individuals, non-spike-specific populations in 7 previously-infected individuals, and EBV-specific populations in 9 individuals. CMV-specific populations were included in the Sheffield dataset only. Boxplots display the median (line), IQR (box), and upper/lower quartile * 1.5 (whiskers).

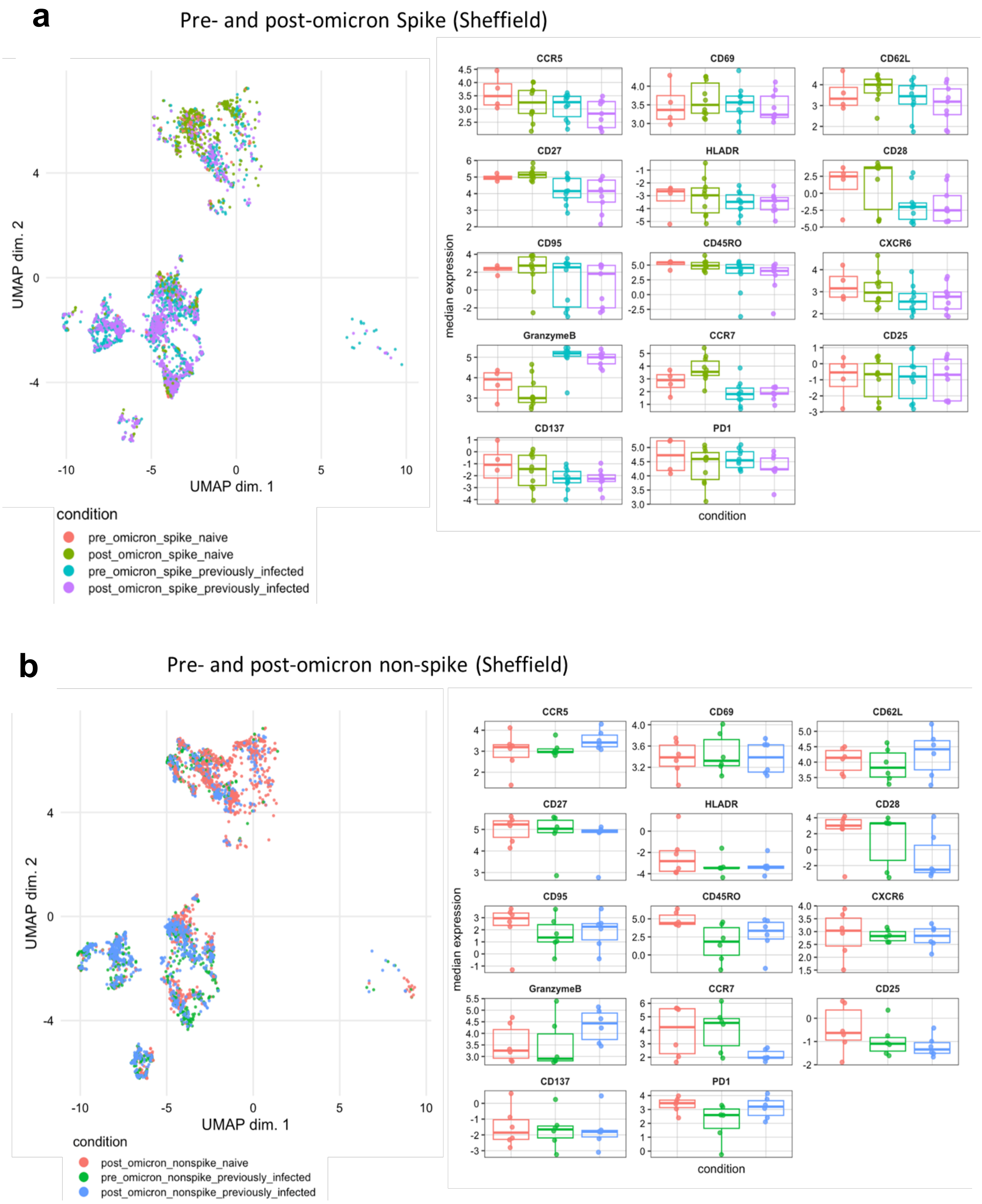

Figure S5. Phenotype of epitope-specific CD8+ T cell populations before and after omicron infection, characterized using multi-dimensional flow cytometry (Sheffield panel). UMAP plots and marker expression levels showing multimer-specific cell clusters from SARS-CoV-2 spike- and non-spike-specific CD8+ T cells using expression of 14

markers. Shown are (**a**) spike-specific populations from 10 naïve and 10 previously-infected individuals; (**b**) non-spike-specific populations from 6 naïve and 6 previously-infected individuals. Boxplots display the median (line), IQR (box), and upper/lower quartile * 1.5 (whiskers).

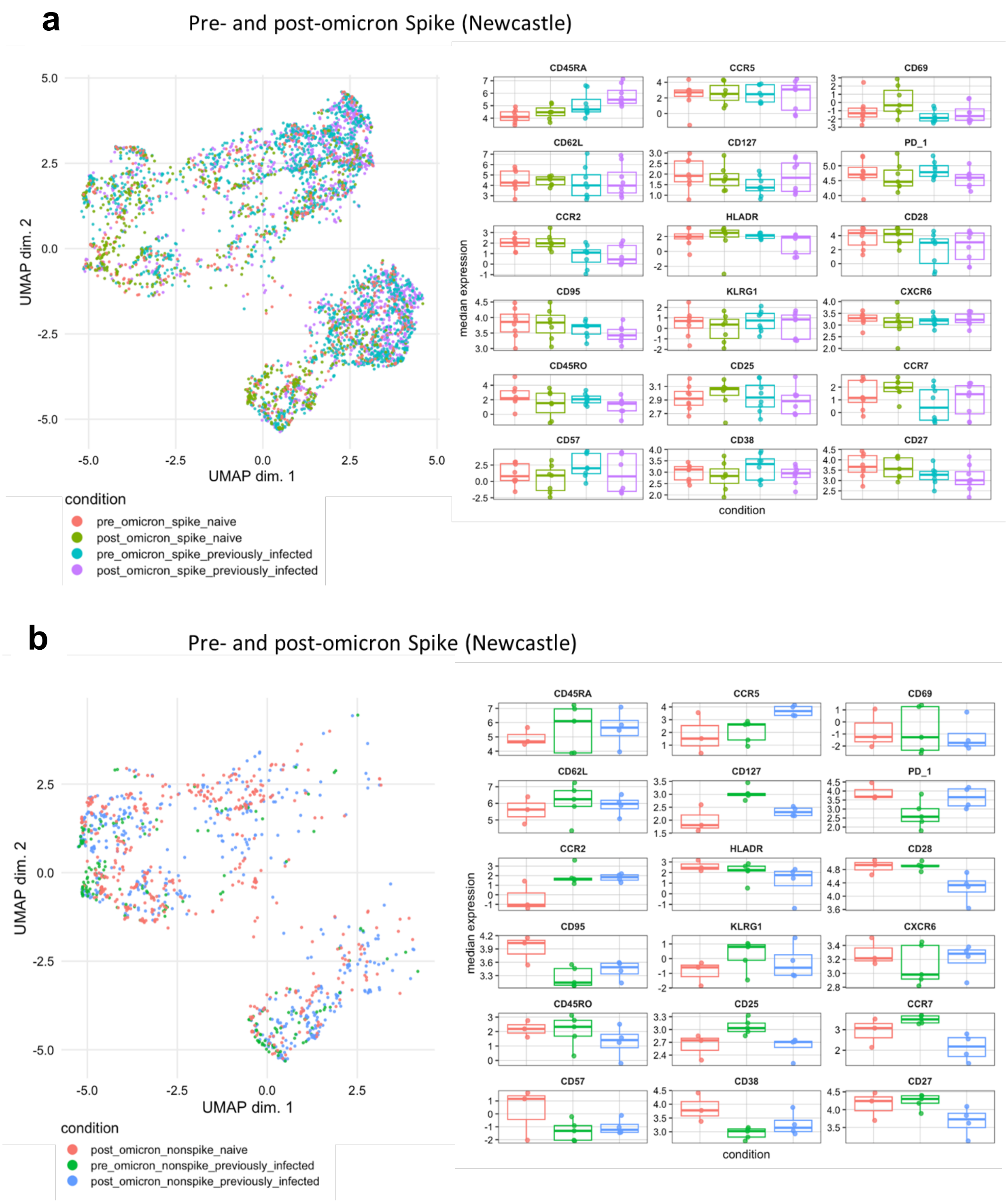

Figure S6. Phenotype of epitope-specific CD8+ T cell populations before and after omicron infection, characterized using multi-dimensional flow cytometry (Newcastle panel). UMAP plots and marker expression levels showing multimer-specific cell clusters from SARS-CoV-2 spike- and non-spike-specific CD8+ T cells using expression of 18 markers. Shown are (**a**) spike-specific populations from 13 naïve and 11 previously-infected

individuals; (**b**) non-spike-specific populations from 5 naïve and 7 previously-infected individuals. Boxplots display the median (line), IQR (box), and upper/lower quartile * 1.5 (whiskers).

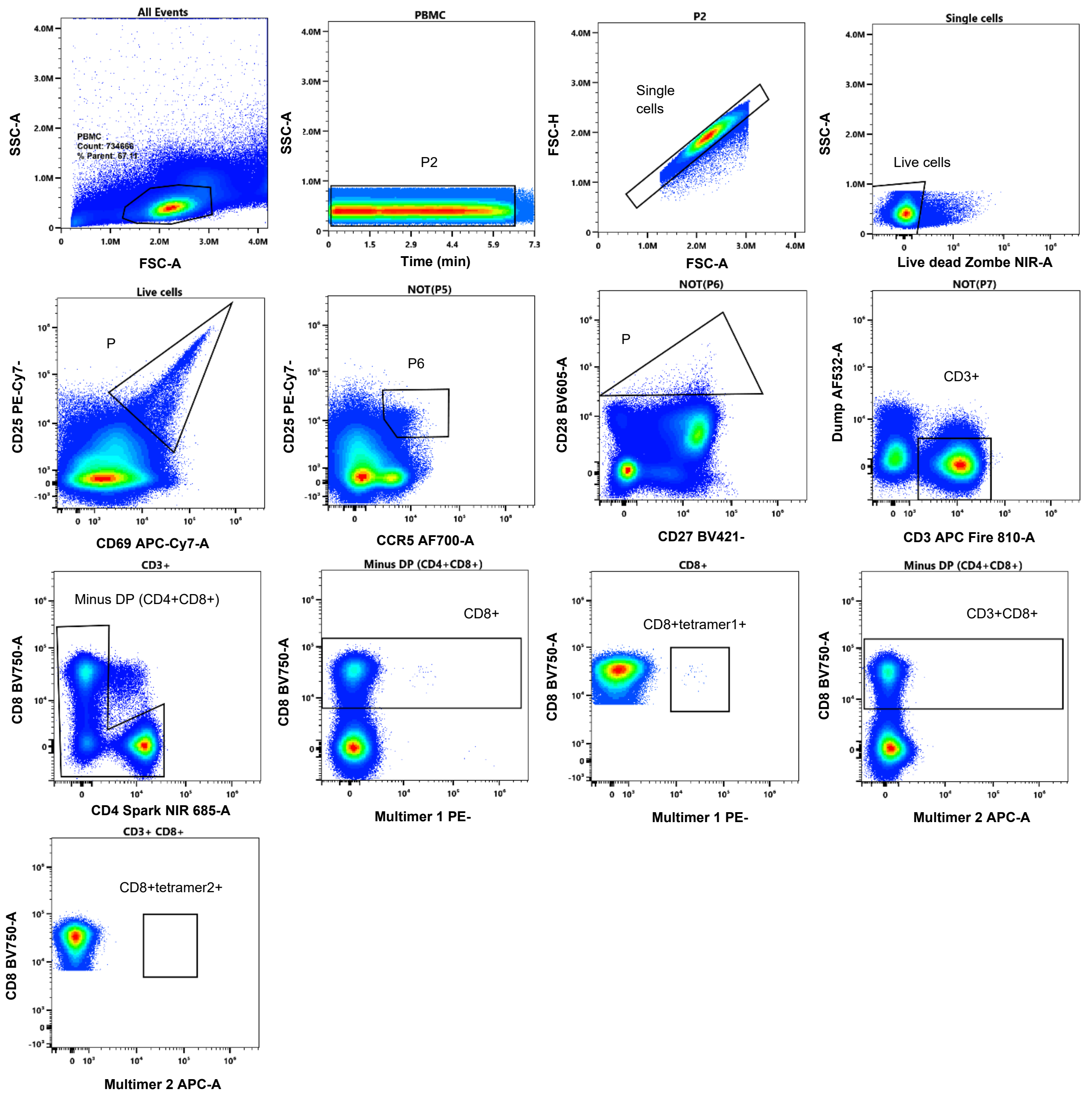

Figure S7. Gating strategy for sorting of multimer-specific CD8+ T cells from participant peripheral blood mononuclear cells. Populations identified by this gating strategy are shown in Figure 6a.

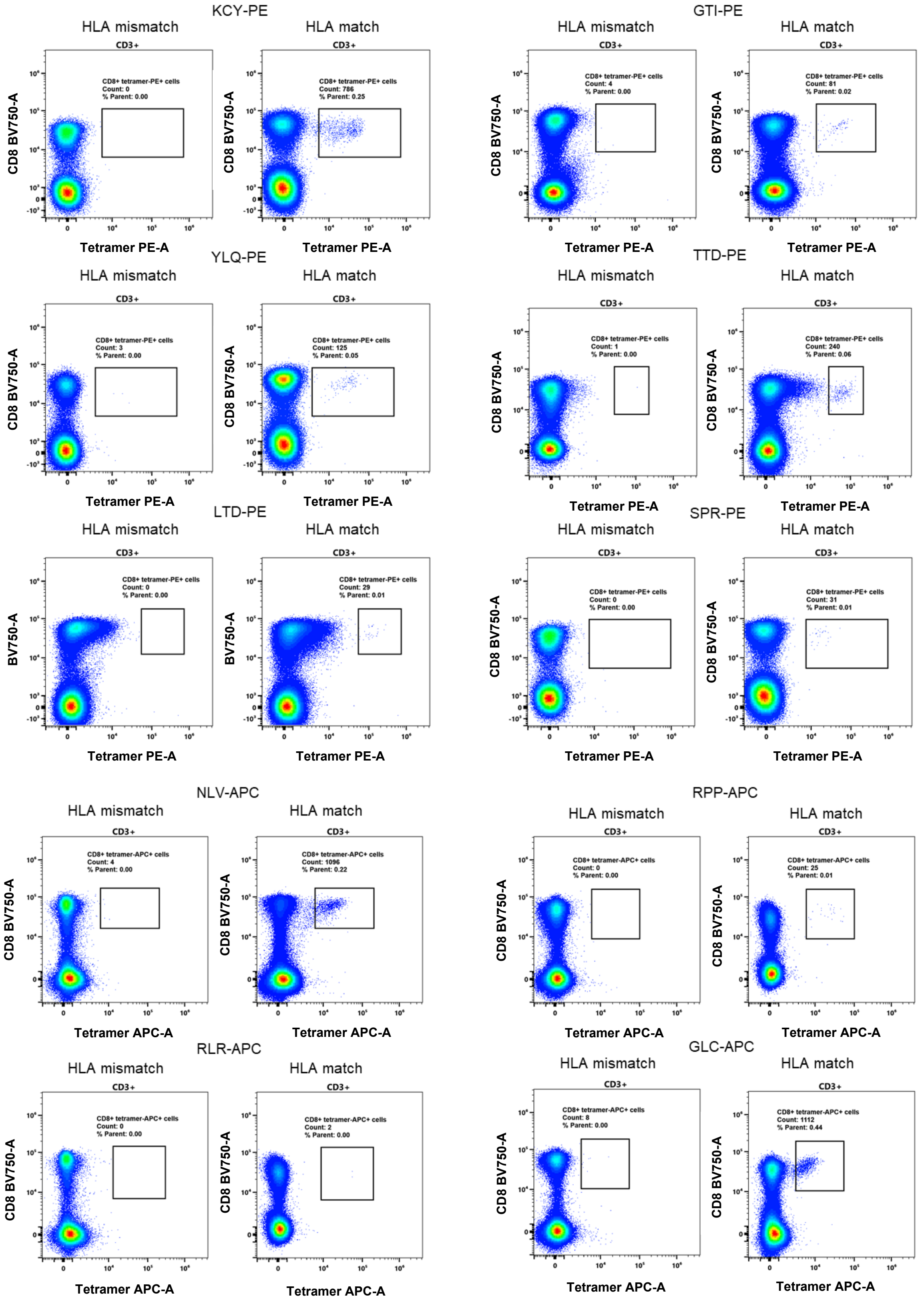

Figure S8. Examples of gating for multimer-specific CD8+ T cells. Shown are multimer-specific populations for all multimers included in the study (PE- or APC- labelled, X axis; CD8- on Y axis, after gating on CD3+ live, singlet cells), alongside staining of peripheral blood mononuclear cells from HLA-mismatched donors.

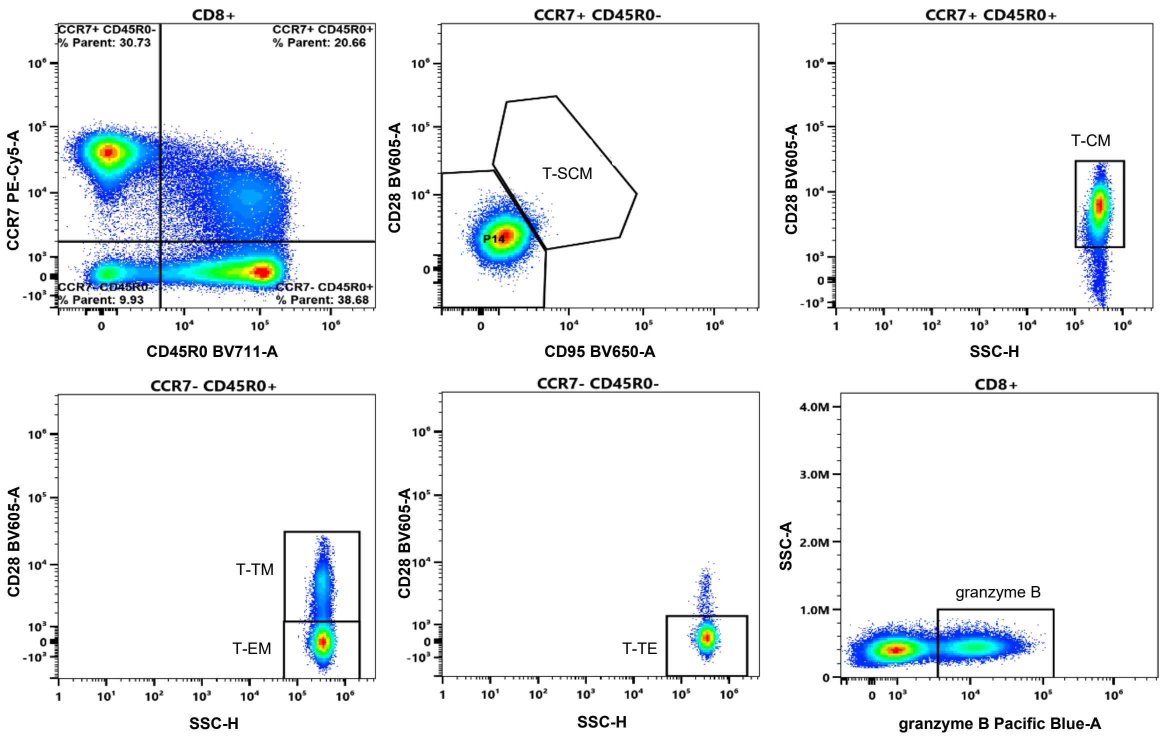

Figure S9. Gating strategy for sorting of CD8+ T cells into memory subsets. The multimer-specific CD8+ population was gated for as shown in Figure S7, and gates were applied to this population to assign cells to memory T cell subsets as shown in Figures 7a-c.

Full list of PITCH consortium authors

First name	Initial	Last name	Institution
Jenna		Ablott	Sheffield Teaching Hospitals NHS Foundation Trust
Priyanka		Abraham	University of Oxford
Sandra		Adele	University of Oxford
Zahra		Ahmed	University of Birmingham
Saly		Al-Taei	University of Birmingham
Mohammad		Ali	University of Oxford
Ali		Amini	University of Oxford
Adrienn		Angyal	University of Sheffield
M.		AzimAnsari	University of Oxford
Rachel		Anslow	University of Oxford
Ana		Atti	UK Health Security Agency
James		Austin	University of Liverpool
Angela		Bailey	Newcastle University
Eleanor		Barnes	University of Oxford
Natalie	A	Barratt	University of Sheffield
Martin		Bayley	University of Sheffield
Sagida		Bibi	University of Oxford
Lucy	H	Booth	University of Cambridge
Alice		Bridges-Webb	University of Oxford
Anthony		Brown	University of Oxford
Rebecca		Brown	University of Sheffield
Holly		Caborn	Sheffield Teaching Hospitals NHS Foundation Trust
Miles		Carroll	University of Oxford
Jeremy		Chalk	University of Oxford
Anu		Chawla	Liverpool University Hospitals NHS Foundation Trust
Elizabeth		Clutterbuck	University of Oxford
Christopher	P	Conlon	University of Oxford
Andrew		Cross	Liverpool University Hospitals NHS Foundation Trust
Debbie		Cross	University of Oxford
Sophie		Davies	University of Oxford
Catherine		de Lara	University of Oxford
Thushan	I	de Silva	University of Sheffield
Alexandra	S	Deeks	University of Oxford
Wanwisa		Dejnirattisai	University of Oxford
Susan	L	Dobson	University of Liverpool
Christina		Dold	University of Oxford
Thomas	M	Drake	University of Edinburgh
Susanna		Dunachie	University of Oxford
Christopher	JA	Duncan	Newcastle University
Elena		Efstathiou	University of Birmingham
David		Eyre	University of Oxford
Alex		Fairman	University of Sheffield
Sian		Faustini	University of Birmingham

Andrew		Filby	Newcastle University
Sarah		Foulkes	UK Health Security Agency
John		Frater	University of Oxford
Lisa		Frending	University of Oxford
Oliver		Galgut	University of Birmingham
Siobhan		Gardiner	University of Oxford
Philip		Goulder	University of Oxford
Jessica		Gregory	Sheffield Teaching Hospitals NHS Foundation Trust
Irina		Grouneva	University of Sheffield
Lotta		Gustafsson	Sheffield Teaching Hospitals NHS Foundation Trust
Carl-Philipp		Hackstein	University of Oxford
Victoria		Hall	UK Health Security Agency
Callum		Halstead	University of Oxford
Sophie		Hambleton	Newcastle University
Muzlifah		Haniffa	Newcastle University
Helen		Hanson	Newcastle University
Alexander		Hargreaves	University of Oxford
Kate		Harrington	Sheffield Teaching Hospitals NHS Foundation Trust
Jenny		Haworth	Newcastle upon Tyne Hospitals Foundation Trust
Carole		Hays	Newcastle University
Luisa	M	Hering	University of Liverpool
Susan		Hopkins	UK Health Security Agency
Emily	C	Horner	University of Cambridge
Hailey		Hornsby	University of Sheffield
Fatima		MariaImlyas	Sheffield Teaching Hospitals NHS Foundation Trust
Jasmin		Islam	UK Health Security Agency
Anni		Jämsén	University of Oxford
Katie		Jeffery	University of Oxford
Sile		Johnson	University of Oxford
Geraldine		Jones	Newcastle University
Mwila		Kasanyinga	University of Oxford
Sinead		Kelly	Newcastle upon Tyne Hospitals Foundation Trust
Maqsood		Khan	Sheffield Teaching Hospitals NHS Foundation Trust
Jon		Kilby	University of Sheffield
Rosemary		Kirk	Sheffield Teaching Hospitals NHS Foundation Trust
Paul		Klenerman	University of Oxford
Barbara		Kronsteiner	University of Oxford
Teresa		Lambe	University of Oxford
Allan		Lawrie	University of Sheffield
Lauren		Lett	University of Liverpool
Chang		Liu	University of Oxford
Stephanie		Longet	University of Oxford
Alison		Lye	Sheffield Teaching Hospitals NHS Foundation Trust
Tom		Malone	University of Oxford
Spyridoula		Marinou	University of Oxford
Chloe		Matthewman	Sheffield Teaching Hospitals NHS Foundation Trust

Philippa	C	Matthews	Francis Crick Institute
David		McDonald	Newcastle University
Jessica		McNeill	Sheffield Teaching Hospitals NHS Foundation Trust
Gracie		Mead	University of Oxford
Naomi		Meardon	Sheffield Teaching Hospitals NHS Foundation Trust
Alexander	J	Mentzer	University of Oxford
Shagun		Misra	Sheffield Teaching Hospitals NHS Foundation Trust
Juthathip		Mongkolsapaya	University of Oxford
Shona	C	Moore	University of Liverpool
Sam	M	Murray	University of Oxford
Isabel		Neale	University of Oxford
Jeremy	M	Nell	Newcastle University
Thomas	AH	Newman	Sheffield Teaching Hospitals NHS Foundation Trust
Alexander	R	Nicols	Newcastle University
Christopher		Norman	Sheffield Teaching Hospitals NHS Foundation Trust
Ane		Ogbe	University of Oxford
Ashley		Otter	UK Health Security Agency
Juyeon		Park	University of Oxford
Brendan	AI	Payne	Newcastle University
Rebecca	P	Payne	Newcastle University
Eloise		Phillips	University of Oxford
Gareth		Platt	University of Liverpool
Megan		Plowright	Sheffield Teaching Hospitals NHS Foundation Trust
Andrew	J	Pollard	University of Oxford
Sonia		Poolan	Newcastle upon Tyne Hospitals Foundation Trust
Nicholas		Provine	University of Oxford
Alex		Richter	University of Birmingham
Chloe		Roddis	Sheffield Teaching Hospitals NHS Foundation Trust
Stefan		Roman	Sheffield Teaching Hospitals NHS Foundation Trust
Leigh		Romaniuk	Newcastle upon Tyne Hospitals Foundation Trust
Patpong		Rongkard	University of Oxford
Sarah	L	Rowland-Jones	University of Sheffield
Ayoub		Saei	UK Health Security Agency
Jose		Schutter	University of Sheffield
Gavin		Screaton	University of Oxford
Adrian		Shields	University of Birmingham
Laura		Silva Reyes	University of Oxford
Donal		Skelly	University of Oxford
Nikki		Smith	University of Sheffield
Jarmila	S	Spegarova	Newcastle University
Lizzie		Stafford	University of Oxford
Gareth		Stephens	Sheffield Teaching Hospitals NHS Foundation Trust
Emily		Stephenson	Newcastle University
Rachel		Stimpson	Sheffield Teaching Hospitals NHS Foundation Trust
Scarlett		Strickland	Sheffield Teaching Hospitals NHS Foundation Trust
Krishanthi		Subramaniam	University of Liverpool

Piyada		Supasa	University of Oxford
Chloe		Tanner	University of Birmingham
Lydia	J	Taylor	Newcastle University
Chitra		Tejpal	University of Oxford
James	ED	Thaventhiran	University of Cambridge
Nicola		Tinker	Sheffield Teaching Hospitals NHS Foundation Trust
Tom		Tipton	University of Oxford
Neal		Townsend	University of Birmingham
Simon		Travis	University of Oxford
Nicola		Trewick	Newcastle University
Stephanie		Tucker	Newcastle University
Aekkachai		Tuekprakhon	University of Oxford
Lance		Turtle	University of Liverpool
Helena		Turton	University of Sheffield
Jessica	K	Tyerman	Newcastle University
Zara		Valiji	University of Oxford
Lisa		Watson	Sheffield Teaching Hospitals NHS Foundation Trust
Rachel		Whitham	Sheffield Teaching Hospitals NHS Foundation Trust
Jayne		Willson	Sheffield Teaching Hospitals NHS Foundation Trust
Barbara		Wilson	Newcastle University
Joseph	D	Wilson	University of Oxford
Steven		Wood	University of Sheffield
Daniel	G	Wootton	University of Liverpool
Amira	AT	Zawia	Sheffield Teaching Hospitals NHS Foundation Trust
Martha		Zewdie	University of Oxford
Peijun		Zhang	University of Sheffield
