# Supplementary material for: Omicron infection following vaccination enhances a broad spectrum of immune responses dependent on infection history
Source: Nat Commun. 2023 Aug 21;14:5065. doi: 10.1038/s41467-023-40592-4 (PMC10442364; doi:10.1038/s41467-023-40592-4)
Supplement: Supplementary file 3 — Reporting Summary [file 41467_2023_40592_MOESM3_ESM.pdf]

nature portfolio

Corresponding author(s): Thushan de Silva

Last updated by author(s): Jun 20, 2023

Reporting Summary

Nature Portfolio wishes to improve the reproducibility of the work that we publish. This form provides structure for consistency and transparency in reporting. For further information on Nature Portfolio policies, see our Editorial Policies and the Editorial Policy Checklist.

Statistics

For all statistical analyses, confirm that the following items are present in the figure legend, table legend, main text, or Methods section.

n/a	Confirmed	
☐	☒	The exact sample size (*n*) for each experimental group/condition, given as a discrete number and unit of measurement
☐	☒	A statement on whether measurements were taken from distinct samples or whether the same sample was measured repeatedly
☐	☒	The statistical test(s) used AND whether they are one- or two-sided<br>*Only common tests should be described solely by name; describe more complex techniques in the Methods section.*
☐	☒	A description of all covariates tested
☐	☒	A description of any assumptions or corrections, such as tests of normality and adjustment for multiple comparisons
☐	☒	A full description of the statistical parameters including central tendency (e.g. means) or other basic estimates (e.g. regression coefficient) AND variation (e.g. standard deviation) or associated estimates of uncertainty (e.g. confidence intervals)
☐	☒	For null hypothesis testing, the test statistic (e.g. *F*, *t*, *r*) with confidence intervals, effect sizes, degrees of freedom and *P* value noted<br>*Give P values as exact values whenever suitable.*
☒	☐	For Bayesian analysis, information on the choice of priors and Markov chain Monte Carlo settings
☒	☐	For hierarchical and complex designs, identification of the appropriate level for tests and full reporting of outcomes
☒	☐	Estimates of effect sizes (e.g. Cohen's *d*, Pearson's *r*), indicating how they were calculated

Our web collection on statistics for biologists contains articles on many of the points above.

Software and code

Policy information about availability of computer code

Data collection

MSD assay results were collected using Discovery Bench 4.0 software provided by MESO SCALE DIAGNOSTICS, LLC.
Flow cytometry data was generated using SpectroFlo® version 3.0.0.
ELISA assay data was generated using HIDEX sense plate reader software version 1.2.1.
ELISpot and FRNT assay data was generated using AID ELISpot software 8.0.

Data analysis

All code and files used to undertake the flow cytometry analysis can be found at https://github.com/RebeccaPPayne/Omicron---Sheffield and https://github.com/RebeccaPPayne/Omicron---Newcastle.
Principal component analysis was performed using open source SIMON software version 0.2.1 (https://genular.org).
Gating analysis of flow cytometry data was performed in SpectroFlo® version 3.0.0.
Multi-dimensional analysis of flow cytometry data was undertaken in R using the packages readxl, CATALYST, cowplot, flowCore, scater, SingleCellExperiment, openxlsx and ggpubr.
Statistical analyses were performed with Graphpad Prism version 9.4.1 and IBM SPSS Software 26.

For manuscripts utilizing custom algorithms or software that are central to the research but not yet described in published literature, software must be made available to editors and reviewers. We strongly encourage code deposition in a community repository (e.g. GitHub). See the Nature Portfolio guidelines for submitting code & software for further information.

Data

Policy information about availability of data

All manuscripts must include a data availability statement. This statement should provide the following information, where applicable:

- Accession codes, unique identifiers, or web links for publicly available datasets
- A description of any restrictions on data availability
- For clinical datasets or third party data, please ensure that the statement adheres to our policy

The complete dataset used in this study has been made publicly available at the Open Science Framework, DOI 10.17605/OSF.IO/9TSZ6 and FACs files and code have been made available at Zenodo https://doi.org/10.5281/zenodo.8045040 and https://doi.org/10.5281/zenodo.8045107

Human research participants

Policy information about studies involving human research participants and Sex and Gender in Research.

Reporting on sex and gender	Previously-infected individuals had a higher proportion of female than male participants (84.2% vs. 58.9%, p=0.01).
Population characteristics	94 individuals from four PITCH sites (Liverpool, Newcastle, Oxford and Sheffield) with SARS-CoV-2 infection occurring after three BNT162b2 mRNA vaccine doses were included in the study, of which 38 (40.4%) had a history of prior SARS-CoV-2 before commencing their vaccine course. The median time from this first infection to the 3rd vaccine dose was 544 days (IQR 514-559), with all but one infection occurring prior to December 2020 when widespread circulation of B.1.1.7/alpha variants occurred (pre-alpha/ancestral). Previously-infected individuals were slightly older than naive healthcare workers (median age 48 vs 41, p=0.02), with a higher proportion of female participants (84.2% vs. 58.9%, p=0.01). All individuals had received their 1st and 2nd vaccine doses a median of 9.6 weeks apart (IQR 8.9-10.9). Omicron infections occurred between 21st December 2021 and 17th May 2022, with sequence data available from 29% of individuals included in the study to confirm SARS-CoV-2 lineage (17 BA.1 and 11 BA.2). Assuming that infections from 20th March 2022 were likely to be caused by BA.2, 62 (66%) infections were classified as probable or confirmed BA.1.<br><br>Our cohort of healthcare workers are relatively young and healthy, and so not entirely representative of the general population. Furthermore, we selected previously-infected individuals who had breakthrough omicron infections within a few months of a 3rd mRNA vaccine dose, and who therefore may not be immunologically similar to those who remained protected for longer. Real-world protection from symptomatic BA.1/BA.2 infection is greater in those with infection followed by three mRNA vaccine doses compared to SARS-CoV-2 naive triple-vaccinated individuals 35, therefore we likely enriched for previously-infected individuals with lower protective immunity.
Recruitment	The PITCH (Protective Immunity from T cells to Covid-19 in Health workers) study is a prospective cohort study of healthcare workers (HCWs) recruited at five sites in the UK (University Hospitals Birmingham NHS Foundation Trust, Liverpool University Hospitals NHS Foundation Trust, Newcastle upon Tyne Hospitals NHS Foundation Trust, Oxford University Hospitals NHS Foundation Trust, and Sheffield Teaching Hospitals NHS Foundation Trust). Eligible participants were adults aged 18 years and over and working as health-care workers (including allied support and laboratory staff). Individuals were recruited by word of mouth, hospital email communications, from hospital-based staff SARS-CoV-2 screening programmes, and enrolling through the wider SIREN study, which PITCH is a substudy thereof. Some participants were recruited under other protocol-aligned REC-approved studies; in Liverpool some participants were recruited under the 'Human immune responses to acute virus infections' study (16/NW/0170). In Oxford, participants were recruited under the GI Biobank Study (16/YH/0247). In Sheffield, participants were recruited under the Observational Biobanking study STHObs (18/YH/0441). All procedures were conducted in accordance with the principles of the Declaration of Helsinki (2008), and the International Conference on Harmonization Good Clinical Practice guidelines. All participants enrolled provided written informed consent.
Ethics oversight	The SIREN study is registered with ISRCTN (Trial ID:252 ISRCTN11041050), and was approved by the Berkshire Research Ethics Committee, Health Research 250 Authority (IRAS ID 284460, REC reference 20/SC/0230), with PITCH recognized as a sub-study on 2 December 2020.<br>The 'Human immune responses to acute virus infections' Study (16/NW/0170) was approved by North West - Liverpool Central Research Ethics Committee on 8 March 2016, and amended on 14th September 2020 and 4th May 2021<br>The GI Biobank Study 16/YH/0247 was approved by the research ethics committee (REC) at Yorkshire & The Humber - Sheffield Research Ethics Committee on 29 July 2016, which has been amended for this purpose on 8 June 2020.

Note that full information on the approval of the study protocol must also be provided in the manuscript.

Field-specific reporting

Please select the one below that is the best fit for your research. If you are not sure, read the appropriate sections before making your selection.

☒ Life sciences ☐ Behavioural & social sciences ☐ Ecological, evolutionary & environmental sciences

For a reference copy of the document with all sections, see nature.com/documents/nr-reporting-summary-flat.pdf

Life sciences study design

All studies must disclose on these points even when the disclosure is negative.

Sample size	Our sample size was based on the number of eligible participants: we included all PITCH participants who at the time of selection had experienced a SARS-CoV-2 omicron infection following three BNT162b2 mRNA vaccine doses, and for whom we had post-dose 3 and post-omicron infections available.
Data exclusions	No data exclusions
Replication	All assays involved appropriate levels of replication as described in the methods for each. FRNT assay was performed in duplicate. Plasma and mucosal antibody assays were performed in duplicate. MSD assays were performed in duplicate. ELISpot assays were performed in duplicate.
Randomization	Allocation to groups was not not random, study participants were defined as either SARS-CoV-2 naive or previously infected at the time of enrolment in the PITCH study based on documented PCR and/or serology from local NHS trusts, or from MSD analysis of S and N plasma IgG levels.
Blinding	This was an observational cohort study with the main groups compared defined by prior infection and vaccination status by the investigators, therefore blinding was not possible.

Reporting for specific materials, systems and methods

We require information from authors about some types of materials, experimental systems and methods used in many studies. Here, indicate whether each material, system or method listed is relevant to your study. If you are not sure if a list item applies to your research, read the appropriate section before selecting a response.

Materials & experimental systems

n/a	Involved in the study	
☐	☒	Antibodies
☐	☒	Eukaryotic cell lines
☒	☐	Palaeontology and archaeology
☒	☐	Animals and other organisms
☒	☐	Clinical data
☒	☐	Dual use research of concern

Methods

n/a	Involved in the study	
☒	☐	ChIP-seq
☐	☒	Flow cytometry
☒	☐	MRI-based neuroimaging

Antibodies

Antibodies used

Reagent (clone), Source, Catalogue number

Flow cytometry

CCR2-BV480 Mouse Anti-Human (LS132.1D9), Becton Dickinson, 747852
CCR5-AF700 Rat Anti-Human (HEK/1/85a), Biolegend, 313713
CCR7-PE-Cy5 Mouse Anti-Human (G043H7), Biolegend, 353272
CCR7-PerCP Mouse Anti-Human (G043H7), BioLegend, 353242
CD127-BUV737 Mouse Anti-Human (HIL-7R-M21), Becton Dickinson, 612794
CD137-PE/Dazzle 594 Mouse Anti-Human (4B4-1), Biolegend, 309826
CD19-Biotin Mouse Anti-Human (HIB19), Biolegend, 302204
CD19-BUV395 Mouse Anti-Human (SJ25C1), Becton Dickinson, 563549
CD25-PE-Cy7 Mouse Anti-Human (M-A251), Becton Dickinson, 557741
CD27-BV421 Mouse Anti-Human (M-T271), Becton Dickinson, 562513
CD27-Super Bright 436 Mouse Anti-Human (0323), ThermoFisher, 62-0279-42
CD28-BV605 Mouse Anti-Human (L293), Becton Dickinson, 742527
CD38-PerCP_ eFluor_710 Mouse Anti-Human (HB7), ThermoFisher, 46-0388-42
CD3-APC Mouse Anti-Human (UCHT1), BioLegend, 300412
CD3-APC/Fire 810 Mouse Anti-Human (SK7), Biolegend, 344858
CD3-PE Mouse Anti-Human (UCHT1), BioLegend, 300408
CD45RO-BV711 Mouse Anti-Human (UCHL1), Becton Dickinson, 563722
CD45RA-Alexa Fluor 647 Mouse Anti-Human (HI100), BioLegend, 304154
CD45RO-Pacific Blue Mouse Anti-Human (UCHL1), BioLegend, 304216
CD4-eFluor 506 Mouse Anti-Human (RPA-T4), ThermoFisher, 69-0049-42
CD4-Spark NIR 685 Mouse Anti-Human (SK3), Biolegend, 344657
CD56-Biotin Mouse Anti-Human (HCD56), Biolegend, 318320
CD57-PerCP_ Cy5.5 Mouse Anti-Human (HNK4), BioLegend, 359622
CD62L-BB515 Mouse Anti-Human (SK11), Becton Dickinson, 565037
CD62L-BB700 Mouse Anti-Human (SK11), Becton Dickinson, 745995

CD69-APC_Cy7 Mouse Anti-Human (FN50), BioLegend, 310914
CD8-BUV805 (SK1), Becton Dickinson, 612889
CD8-BV750 Mouse Anti-Human (SK1), Biolegend, 344756
CD95-BV650 Mouse Anti-Human (DX2), Becton Dickinson, 740589
CXCR6-BV786 Mouse Anti-Human (13B 1E5), Becton Dickinson, 743602
Granzyme B-Pacific Blue Mouse Anti-Human (GB11), Biolegend, 515408
HLA-DR-BV570 Mouse Anti-Human (L243), Biolegend, 307638
KLRG1-BV711 Hamster Anti-Human (2F1), BioLegend, 138427
PD-1-BV421 Mouse Anti-Human (EH12.1), Becton Dickinson, 562516
PD-1-VioBright FITC Mouse Anti-Human (PD 1.3.1.3.), Miltenyi Biotec, 130-117-681
Streptavidin AF532, ThermoFisher, S11224
Zombie NIR, Biolegend, 423106

FRNT assay
Goat Anti-Human IgG (Fc specific)-HRP (polyclonal), Sigma, A0170
Human anti-N mAb (mAb206), not available commercially

N-specific ELISA
Goat anti-Human IgG (Gamma chain) Cross-Adsorbed Secondary Antibody-HRP (polyclonal), Invitrogen, 62-8420

sIgA ELISA
IgA from human colostrum (polyclonal), Sigma, I2636
Mouse Anti-Human IgA secretory component (HP6141), Calbiochem, 411423
Goat Anti-Mouse IgG Fc Cross-Adsorbed Secondary Antibody-HRP (polyclonal), Invitrogen, 31439
Goat Anti-Human Kappa-UNLB (polyclonal), Southern Biotech, 2060-01

Goat Anti-Human Lambda-UNLB (polyclonal), Southern Biotech, 2070-01

Validation

CCR2-BV480 Mouse Anti-Human (LS132.1D9), Becton Dickinson, 747852 https://www.bdbiosciences.com/en-gb/products/reagents/flow-cytometry-reagents/research-reagents/single-color-antibodies-ruo/bv480-mouse-anti-human-ccr2-cd192.747852

CCR5-AF700 Rat Anti-Human (HEK/1/85a), Biolegend, 313713 https://www.biolegend.com/en-us/search-results/alexa-fluor-700-anti-human-cd195-ccr5-antibody-3438

CCR7-PE-Cy5 Mouse Anti-Human (G043H7), Biolegend, 353272 https://www.biolegend.com/en-gb/products/pe-cyanine5-anti-human-cd197-ccr7-antibody-21177

CCR7-PerCP Mouse Anti-Human (G043H7), BioLegend, 353242 https://www.biolegend.com/en-gb/products/percp-anti-human-cd197-ccr7-antibody-10712

CD127-BUV737 Mouse Anti-Human (HIL-7R-M21), Becton Dickinson, 612794 https://www.bdbiosciences.com/en-gb/products/reagents/flow-cytometry-reagents/research-reagents/single-color-antibodies-ruo/buv737-mouse-anti-human-cd127.612794

CD137-PE/Dazzle 594 Mouse Anti-Human (4B4-1), Biolegend, 309826 https://www.biolegend.com/en-us/products/pe-dazzle-594-anti-human-cd137-4-1bb-antibody-12280

CD19-Biotin Mouse Anti-Human (HIB19), Biolegend, 302204 https://www.biolegend.com/ja-jp/products/biotin-anti-human-cd19-antibody-716

CD19-BUV395 Mouse Anti-Human (SJ25C1), Becton Dickinson, 563549 https://www.bdbiosciences.com/en-eu/products/reagents/flow-cytometry-reagents/research-reagents/single-color-antibodies-ruo/buv395-mouse-anti-human-cd19.563549

CD25-PE-Cy7 Mouse Anti-Human (M-A251), Becton Dickinson, 557741 https://www.bdbiosciences.com/en-gb/products/reagents/flow-cytometry-reagents/research-reagents/single-color-antibodies-ruo/pe-cy-7-mouse-anti-human-cd25.557741

CD27-BV421 Mouse Anti-Human (M-T271), Becton Dickinson, 562513 https://www.bdbiosciences.com/en-us/products/reagents/flow-cytometry-reagents/research-reagents/single-color-antibodies-ruo/bv421-mouse-anti-human-cd27.562513

CD27-Super Bright 436 Mouse Anti-Human (0323), ThermoFisher, 62-0279-42 https://www.thermofisher.com/antibody/product/CD27-Antibody-clone-O323-Monoclonal/62-0279-42

CD28-BV605 Mouse Anti-Human (L293), Becton Dickinson, 742527 https://www.bdbiosciences.com/en-at/products/reagents/flow-cytometry-reagents/research-reagents/single-color-antibodies-ruo/bv605-mouse-anti-human-cd28.742527

CD38-PerCP_ eFluor_710 Mouse Anti-Human (HB7), ThermoFisher, 46-0388-42 https://www.thermofisher.com/antibody/product/CD38-Antibody-clone-HB7-Monoclonal/46-0388-42

CD3-APC Mouse Anti-Human (UCHT1), BioLegend, 300412 https://www.biolegend.com/en-us/products/apc-anti-human-cd3-antibody-861?GroupID=BLG5900

CD3-APC/Fire 810 Mouse Anti-Human (SK7), Biolegend, 344858 https://www.biolegend.com/nl-be/products/apc-fire-810-anti-human-cd3-antibody-19515

CD3-PE Mouse Anti-Human (UCHT1), BioLegend, 300408 https://www.biolegend.com/en-gb/productstab/pe-anti-human-cd3-antibody-865

CD45RO-BV711 Mouse Anti-Human (UCHL1), Becton Dickinson, 563722 https://www.bdbiosciences.com/en-eu/products/reagents/flow-cytometry-reagents/research-reagents/single-color-antibodies-ruo/bv711-mouse-anti-human-cd45ro.563723

CD45RA-Alexa Fluor 647 Mouse Anti-Human (HI100), BioLegend, 304154 https://www.biolegend.com/en-us/soluble-mhc/alexa-fluor-647-anti-human-cd45ra-antibody-3338?GroupID=GROUP658

CD45RO-Pacific Blue Mouse Anti-Human (UCHL1), BioLegend, 304216 https://www.biolegend.com/en-gb/products/pacific-blue-anti-human-cd45ro-antibody-3342

CD4-eFluor 506 Mouse Anti-Human (RPA-T4), ThermoFisher, 69-0049-42 https://www.thermofisher.com/antibody/product/CD4-Antibody-clone-RPA-T4-Monoclonal/69-0049-42

CD4-Spark NIR 685 Mouse Anti-Human (SK3), Biolegend, 344657 https://www.biolegend.com/en-us/products/spark-nir-685-anti-human-cd4-antibody-18516?GroupID=GROUP28

CD56-Biotin Mouse Anti-Human (HCD56), Biolegend, 318320 https://www.biolegend.com/nl-be/products/biotin-anti-human-cd56-ncam-antibody-4076

CD57-PerCP_ Cy5.5 Mouse Anti-Human (HNK4), BioLegend, 359622 https://www.biolegend.com/en-us/products/percp-cyanine5-5-anti-human-cd57-antibody-12127?GroupID=BLG8483

CD62L-BB515 Mouse Anti-Human (SK11), Becton Dickinson, 565037 https://www.bdbiosciences.com/en-be/products/reagents/flow-cytometry-reagents/research-reagents/single-color-antibodies-ruo/bb515-mouse-anti-human-cd62l.565037

CD62L-BB700 Mouse Anti-Human (SK11), Becton Dickinson, 745995 https://www.bdbiosciences.com/zh-cn/products/reagents/flow-cytometry-reagents/research-reagents/single-color-antibodies-ruo/BB700-Mouse-Anti-Human-CD62L.745995

CD69-APC_Cy7 Mouse Anti-Human (FN50), BioLegend, 310914 https://www.biolegend.com/nl-nl/products/apc-cyanine7-anti-human-cd69-antibody-1917

CD8-BUV805 (SK1), Becton Dickinson, 612889 https://www.bdbiosciences.com/en-us/products/reagents/flow-cytometry-reagents/research-reagents/single-color-antibodies-ruo/buv805-mouse-anti-human-cd8.612889

CD8-BV750 Mouse Anti-Human (SK1), Biolegend, 344756 https://www.biolegend.com/en-us/products/brilliant-violet-750-anti-human-cd8-antibody-17792?pdf=true&displayInline=true&leftRightMargin=15&topBottomMargin=15&filename=Brilliant%20Violet%20750%E2%84%A2%20anti-human%20CD8%20Antibody.pdf&v=20221230073200

CD95-BV650 Mouse Anti-Human (DX2), Becton Dickinson, 740589 https://www.bdbiosciences.com/en-au/products/reagents/flow-cytometry-reagents/research-reagents/single-color-antibodies-ruo/bv650-mouse-anti-human-cd95.740589

CXCR6-BV786 Mouse Anti-Human (13B 1E5), Becton Dickinson, 743602 https://www.bdbiosciences.com/en-be/products/reagents/flow-cytometry-reagents/research-reagents/single-color-antibodies-ruo/bv786-mouse-anti-human-cxcr6-cd186.743602

Granzyme B-Pacific Blue Mouse Anti-Human (GB11), Biolegend, 515408 https://www.biolegend.com/ja-jp/products/pacific-blue-anti-human-mouse-granzyme-b-antibody-8612

HLA-DR-BV570 Mouse Anti-Human (L243), Biolegend, 307638 https://www.biolegend.com/en-gb/antibodies-and-more/brilliant-violet-570-anti-human-hla-dr-antibody-7457

KLRG1-BV711 Hamster Anti-Human (2F1), BioLegend, 138427 https://www.biolegend.com/nl-nl/products/brilliant-violet-711-anti-mouse-human-klrg1-mafa-antibody-13583

PD-1-BV421 Mouse Anti-Human (EH12.1), Becton Dickinson, 562516 https://www.bdbiosciences.com/en-gb/products/reagents/flow-cytometry-reagents/research-reagents/single-color-antibodies-ruo/bv421-mouse-anti-human-cd279-pd-1.565935

PD-1-VioBright FITC Mouse Anti-Human (PD 1.3.1.3.), Miltenyi Biotec, 130-117-681 https://www.miltenyibiotec.com/GB-en/products/cd279-pd1-antibody-anti-human-pd1-3-1-3.html#conjugate=vio-bright-fitc:size=100-tests-in-200-ul

Streptavidin AF532, ThermoFisher, S11224 https://www.thermofisher.com/order/catalog/product/S11224

Zombie NIR, Biolegend, 423106 https://www.biolegend.com/en-us/products/zombie-nir-fixable-viability-kit-8657?GroupID=BLG2181

Goat Anti-Human IgG (Fc specific)-HRP (polyclonal), Sigma, A0170 https://www.sigmaaldrich.com/GB/en/product/sigma/a0170?gclid=Cj0KCQjw7aqkBhDPARIsAKGa0oJoMdvgiPewgggqHCe5yh1Ai4_gbDYFZDYOA57vNdEP12Bff_ofVwQaAoRJEALw_wcB&gclsrc=aw.ds

Human anti-N mAb (mAb206) is not a commercial antibody, it wasidentified and produced in-house and is described in Dejnirattisai et al., 2021, Cell 184, 2183–2200, https://doi.org/10.1016/j.cell.2021.02.032

N-specific ELISA
Goat anti-Human IgG (Gamma chain) Cross-Adsorbed Secondary Antibody-HRP (polyclonal), Invitrogen, 62-8420 https://www.thermofisher.com/antibody/product/Goat-anti-Human-IgG-Gamma-chain-Cross-Adsorbed-Secondary-Antibody-Polyclonal/62-8420

IgA from human colostrum (polyclonal), Sigma, I2636 https://www.sigmaaldrich.com/GB/en/product/sigma/i2636

Mouse Anti-Human IgA secretory component (HP6141), Calbiochem, 411423 https://www.merckmillipore.com/GB/en/product/Mouse-Anti-Human-IgA-Secretory-HP6141,EMD_BIO-411423#anchor_PDS

Goat Anti-Mouse IgG Fc Cross-Adsorbed Secondary Antibody-HRP (polyclonal), Invitrogen, 31439 https://www.thermofisher.com/order/genome-database/dataSheetPdf?producttype=antibody&productsubtype=antibody_secondary&productId=31439&version=310

Goat Anti-Human Kappa-UNLB (polyclonal), Southern Biotech, 2060-01 https://www.southernbiotech.com/goat-anti-human-kappa-unlb-2060-01

Goat Anti-Human Lambda-UNLB (polyclonal), Southern Biotech, 2070-01 https://www.southernbiotech.com/goat-anti-human-lambda-unlb-2070-01

Eukaryotic cell lines

Policy information about cell lines and Sex and Gender in Research

Cell line source(s)	Vero cells from ATCC (Cat#CCL-81)
Authentication	Not authenticated
Mycoplasma contamination	Mycoplasma contamination - not detected by manufacturer
Commonly misidentified lines (See ICLAC register)	None used in this study

Flow Cytometry

Plots

Confirm that:

☒ The axis labels state the marker and fluorochrome used (e.g. CD4-FITC).

☒ The axis scales are clearly visible. Include numbers along axes only for bottom left plot of group (a 'group' is an analysis of identical markers).

☒ All plots are contour plots with outliers or pseudocolor plots.

☒ A numerical value for number of cells or percentage (with statistics) is provided.

Methodology

Sample preparation

PBMCs were isolated from heparinised whole blood taken from PITCH participants via density gradient centrifugation. Blood was added to Leucosep tubes prefilled with Lymphoprep, and spun at 1000g for 10 minutes at room temperature (acceleration = 2, brake = 0). PBMCs were collected, washed with RPMI and spun down at 400g for 5 minutes. The pellet was resuspended in 5 mL red blood cell lysis buffer (Qiagen; 158902) for 5 minutes before washing with RPMI and spun down at 400g for 5 minutes. Cells were counted and frozen down in an FBS + 10% DMSO solution, and kept at -196°C in liquid nitrogen storage assays were performed.

For flow experiments, cryopreserved PBMCs were rapidly thawed and rested for 2-4 hours in R10 media (RPMI + 10% FBS + 1% pen/strep) at 37°C. After resting and prior to staining, cells were washed in PBS and divided into FACS tubes at 2-3 x 106 cells per sample. Firstly, samples and HLA mismatched negative control donors were stained with PE conjugated SARS-CoV-2 spike or non-spike specific MHC pentamer/dextramers (PE-HLA-A*03:01 KCYGVSPTK S378, ProImmune, peptide code 4443, PE-HLA-A*02:01 YLQPRTFLL S269, PE-HLA-A*01:01 LTDEMIAQY S865, PE-HLA-B*57:01 GTITSGWTF S879, PE-HLA-A*01:01 TTDPSSFLGRY RP1637, PE-HLA-B*07:02 SPRWYFYYL NCP105, Table S3.) and APC conjugated EBV and CMV pentamer/dextramers (APC-HLA-A*02:01 GLCTLVAML BMLF-1259, APC-HLA-B*07:02-RPPIFIRRL EBNA-3A247, APC-HLA-A*03:01 RLRAEQVK EBNA-3A603 or APC-HLA-A*02:01 NLVPMVATV CMV pp65495, Table S3.) for 15 minutes at 37°C. Surrogate CD3 conjugates for PE and APC were used as reference controls for unmixing either by staining cells (Newcastle) or beads (Sheffield). After pentamer/dextramer staining, samples were washed in PBS and re-suspended in the residual volume (~50μl). Samples were stained with Live/Dead Zombie NIR (Biolegend, 423106) for 10 minutes at RT, after which and without washing, the surface antibody cocktail diluted in Brilliant Stain Buffer (Becton Dickinson, 563794) was added for a further 20 minutes. Single stain reference controls were stained as per Tables S1 and S2 on 2-5 x 105 cells/mL or UltraComp eBeads™ (ThermoFisher, 01-2222-42). The Live/Dead Zombie NIR reference control was prepared by staining a 50/50 mixture of live and heat inactivated (56°C, 7 minutes) PBMCs. Following the extracellular staining, samples prepared in Sheffield were also stained for granzyme B using the BD Cytofix/Cytoperm™ Kit (Cat. No. 554714) according to the manufacturer's instructions. Samples and reference controls in Newcastle were washed in PBS after extracellular staining and fixed for 20 minutes in 2% formaldehyde at RT. After fixation, samples were washed in FACS buffer and re-suspended in an appropriate volume for acquisition on a CyTEK AURORA 3L (Sheffield) or 5L (Newcastle) system.

Instrument

CyTEK AURORA 3 laser system, CyTEK AURORA 5 laser system

Software

Gating analysis of flow cytometry data was performed in SpectroFlo® (version 3.0.0). Statistical analysis was performed in Graphpad Prism version 9.4.

Cell population abundance

There was no cell sorting included in the experiments.

Gating strategy

Cell debris and neutrophil contamination were excluded by placing a gate onto the PBMCs on an FSC/SSC plot. We used an SSC/time plot to eliminate any issues with acquisition. Doublets were then removed by using an FSC-H/FSC-A plot and live cells were identified based on their lack of staining with the live/dead Zombie NIR dye. CD56+ and CD19+ cells were excluded and the CD56-/CD19-/CD3+ cells were selected for further analysis. The multimer-binding CD3+/CD8+ cells were identified by using a CD8/Multimer-PE or CD8/Multimer-APC dot plot and the gates were applied based on the staining of HLA-mismatched samples.

☒ Tick this box to confirm that a figure exemplifying the gating strategy is provided in the Supplementary Information.
